# Different cervical cancer screening approaches in a Chinese multicentre study

**DOI:** 10.1038/sj.bjc.6604840

**Published:** 2009-01-06

**Authors:** N Li, J-F Shi, S Franceschi, W-H Zhang, M Dai, B Liu, Y-Z Zhang, L-K Li, R-F Wu, H De Vuyst, M Plummer, Y-L Qiao, G Clifford

**Affiliations:** 1 International Agency for Research on Cancer, 150 cours Albert Thomas, 69372 Lyon cedex 08, France; 2 Cancer Institute of the Chinese Academy of Medical Sciences, 17 South Pan Jia Yuan LN, PO Box 2258, Beijing 100021, China; 3 Shanxi Provincial Tumor Hospital, No 3, Zhigongxincun, Changcheng District, 030013 Taiyuan, Shanxi Province, China; 4 Liaoning Provincial Tumor Hospital, 44 Xiaoheyen Road, Shenyang 110042, China; 5 Shenzhen Hospital of Beijing University, 1120 Lianhua Road, Futian, Shenzhen City 518036, Guangdong Province, China

**Keywords:** cervical cancer, screening, human papillomavirus, China

## Abstract

To evaluate alternative cervical cancer screening methods, digital colposcopy and collection of cervical exfoliated cells for liquid-based cytology (LBC) and hybrid capture 2 (HC2) testing were performed among 2562 women aged 15–59 years in three study sites in the People's Republic of China (rural Shanxi province, Shenyang city in Liaoning province and Shenzhen city in Guangdong province). Visual inspection with acetic acid (VIA) was also evaluated independently from colposcopy. A total of 74 cases of histologically confirmed cervical intraepithelial neoplasia grade 2 or worse (CIN2+) were identified, and 16 CIN2+ cases were imputed among unbiopsied women to correct for verification bias. Corrected sensitivity for CIN2+ was 37% for VIA, 54% for colposcopy, 87% for LBC with a threshold of atypical cells of undetermined significance (LBC⩾ASCUS), 90% for HC2, 84% for LBC using HC2 to triage ASCUS and 96% for positivity to LBC⩾ASCUS or HC2. For VIA, sensitivity was much lower among women ⩾40 years (12%) than those aged ⩽39 years (50%). Specificity varied from 77% for positivity to LBC⩾ASCUS or HC2, up to 94% for LBC using HC2 to triage ASCUS. In conclusion, LBC, HC2 and their combinations performed well, whereas VIA missed a majority of CIN2+, particularly in older women. Digital colposcopy performed better than VIA, but still missed nearly half of CIN2+ in this study.

Important reductions in cervical cancer incidence have been seen in developed countries; thanks to cytological screening (Peto *et al*, 2004). However, requirements for well-trained personnel and the need for repeated visits have made cytological screening expensive and logistically difficult to implement in low-resource settings (Kitchener *et al*, 2006), where more than 80% of cervical cancer cases worldwide currently occur (Parkin and Bray, 2006).

During the last decade, alternatives to cytological screening have been evaluated in low-resource settings (Belinson *et al*, 2001; Ferreccio *et al*, 2003; De Vuyst *et al*, 2005; IARC, 2005; Sarian *et al*, 2005; Sangwa-Lugoma *et al*, 2006; Almonte *et al*, 2007; Pretorius *et al*, 2007; Sankaranarayanan *et al*, 2007). Visual inspection with acetic acid (VIA) is a low-cost option that allows the possibility of screening and treatment in one visit (Denny *et al*, 2005), and has been shown to significantly reduce cervical cancer mortality in a large randomised controlled trial (Sankaranarayanan *et al*, 2007). Furthermore, shifting of screening towards the detection of human papillomavirus (HPV), the main cause of cervical cancer, is increasingly being considered (Denny *et al*, 2000; Belinson *et al*, 2001; Sankaranarayanan *et al*, 2004b; De Vuyst *et al*, 2005; Sarian *et al*, 2005; Cuzick *et al*, 2006; Almonte *et al*, 2007; Mayrand *et al*, 2007).

We, therefore, nested an evaluation of the performance and variability of various screening approaches among three population-based samples of women from the People's Republic of China invited to undergo gynaecological examination within the framework of the International Agency for Research on Cancer (IARC) HPV Prevalence Surveys.

## Materials and methods

### Study subjects

Between March 2004 and July 2005, the Cancer Institute of the Chinese Academy of Medical Sciences (CI-CAMS) and the IARC carried out three population-based surveys of HPV prevalence among women aged 15–59 years in China, namely in rural Shanxi Province (Dai *et al*, 2006); Shenyang City, Liaoning Province (Li *et al*, 2006) and Shenzhen City, Guangdong Province (Wu *et al*, 2007). Complete descriptions of the sample populations including sociodemographic, sexual and reproductive factors have been reported previously (Dai *et al*, 2006; Li *et al*, 2006; Wu *et al*, 2007). Participation rates were 56% in Shanxi (Dai *et al*, 2006), 72% in Liaoning (Li *et al*, 2006) and 76% in Guangdong (Wu *et al*, 2007).

All participants signed an informed consent form according to the recommendations of the IARC and CI-CAMS ethical review committees, which approved the study.

Among the 2600 participants that underwent a gynaecological examination, 28 women with hysterectomy, one with missing VIA and colposcopy results and nine with missing HC2 results were excluded, leaving 2562 eligible women (743 from Shanxi, 699 from Liaoning and 1120 from Guangdong) for the present analysis.

### Collection of cervical cells

All participants in our study underwent a gynaecological examination performed by a gynaecologist. A cytobrush was inserted into the endocervical canal and rotated gently to collect cells from the endo- and ectocervix. The brush containing cellular material was then placed in a vial containing CytoRich transport media (AutoCyte, Tripath Imaging, Burlington, NC, USA), to be used to perform both liquid-based cytology (LBC) and HPV testing.

Human papillomavirus genotyping has been reported previously (Dai *et al*, 2006; Li *et al*, 2006; Wu *et al*, 2007).

### Screening tests

Liquid-based cytology, HC2 and digital colposcopy were performed in all three study sites, completely independently from each other (i.e., each was performed by a different gynaecologist who was blinded to all other test results). Visual inspection with acetic acid was also performed everywhere, but only in Liaoning and Guangdong was it performed by a different gynaecologist than digital colposcopy. In Shanxi, it was performed by the same gynaecologist as colposcopy, and hence was not evaluated (Table 1).

In Shanxi and Liaoning, there was also an *ad hoc* evaluation of an optical device employing fluorescence spectroscopy technology. It was used to take readings in each quadrant of the cervix of all women before VIA, with positive diagnoses determined automatically by the device's algorithm. Fluorescence spectroscopy showed very poor sensitivity (18.3%) and specificity (79.9%; YL Qiao, personal communication), and is not formally evaluated in this report. However, findings from biopsies prompted by fluorescence spectroscopy-positive examinations were used to reduce verification bias.

#### VIA

The cervix was inspected with the naked eye using a speculum and a bright halogen focus lamp 1 min after application of 5% acetic acid. The VIA result was reported by quadrant, as per the criteria in the IARC manual (Sankaranarayanan and Wesley, 2003). Visual inspection with acetic acid was classified as negative when no acetowhite lesions were seen, or when only ill-defined, scattered or geographic acetowhite lesions away from the squamocolumnar junction were detected. Visual inspection with acetic acid was classified as positive when dense, opaque, well-defined acetowhite lesions touching the squamocolumnar junction or cervical growths turning acetowhite were seen (Sankaranarayanan and Wesley, 2003).

#### Digital colposcopy

All women underwent digital colposcopic assessment (Digital Video Colposcope SLC-2000, Shenzhen Goldway Industrial Inc., Shenzhen, China) as a primary screening test, performed by assessing abnormal areas after application of acetic acid (Sellors and Sankaranarayanan, 2003). Observations were recorded by quadrant.

#### LBC

Liquid-based cytology was performed either at CI-CAMS, Beijing, China (for Shanxi and Liaoning), or the Shenzhen Hospital of Beijing University, Shenzhen, China (for Guangdong). Slides were prepared from the CytoRich-preserved specimen according to the manufacturer's standard protocol, and results were classified according to the Bethesda System (Wright *et al*, 2002). All abnormal smears as well as 10% of normal smears chosen at random were reviewed by a second experienced cytologist.

#### HC2

Second-generation hybrid capture microplate-based HPV testing (HC2 test; Digene Corporation, Gaithersburg, MD, USA) was performed in CI-CAMS, using the same samples as for LBC, according to the manufacturer's instructions. The test uses an RNA probe mixture of 13 high-risk HPV types. The manufacturer-recommended cutoff of 1.0 pg ml^−1^ was used to define samples as HPV-positive.

### Cervical disease assessment

Colposcopy was used during the first screening visit to obtain a biopsy from the suspicious area of all women with abnormal findings by VIA, colposcopy and/or fluorescence spectroscopy.

Colposcopy was also subsequently used to obtain biopsies from all women with low-grade squamous intraepithelial lesions (LSILs) or worse, or those with HC2-positive atypical squamous cells of undetermined significance (ASCUS), but without biopsy in the first visit, when these results became available (approximately one month later). In the absence of a specific suspicious area, biopsies were taken from each quadrant of the transformation zone. Women whose entire squamocolumnar junction could not be seen upon colposcopy underwent endocervical curettage.

Cervical biopsies were prepared and read by a pathologist at CI-CAMS, Beijing, China. In this study, cervical abnormalities were defined as the presence of histologically confirmed cervical intraepithelial neoplasia grade 2 or worse (CIN2+). Treatment of colposcopy-detected lesions was performed according to local protocols, primarily using loop electrosurgical excision procedures.

A total of 653 biopsies were obtained, 195 of which were triggered exclusively by positive findings at fluorescence spectroscopy and 458 of which were indicated by positive findings at one or more of the four screening approaches assessed in this study. Among these colposcopy-directed biopsies, 74 cases of CIN2+ (11.3%) were diagnosed (including 51 CIN2, 22 CIN3 and one cancer).

### Statistical analysis

Conventional screening indices, including sensitivity, specificity, positive predictive value (PPV), negative predictive value (NPV) and their 95% confidence intervals (CIs) were calculated in two ways: first, ‘crude’ indices using only histologically confirmed CIN2+ and assuming that all 1909 women without a biopsy were histolgically negative; and second, ‘corrected’ indices after imputation of the missing data (Almonte *et al*, 2007). Observations on women with missing biopsies were replaced by pseudo-observations with each possible value of the missing diagnosis, and weighted by the estimated probability of having that diagnosis given the screening test results (Appendix 1). Robust standard errors were used to account for the uncertainty in the imputation (White, 1982). The Pearson *χ*^2^-test and corresponding *P*-values were used to calculate eventual heterogeneity of test performance by age and centre.

## Results

Selected characteristics of the three study populations are described in Table 1. Overall, 54.3% of women were younger than 40 years old, 50.7% had received secondary education, 9.9% were unmarried, 16.1% had more than one lifetime sexual partner and 13.5% reported an earlier Pap smear.

Screening test positivity is also shown in Table 1 and, overall, was 11.4% for VIA, 16.2% for colposcopy, 17.1% for LBC with a threshold of ASCUS (LBC⩾ASCUS) and 16.3% for HC2. Biopsies were taken among 40.5, 33.2 and 10.7% of participants in Shanxi, Liaoning and Guangdong, respectively (Table 1).

### Performance of the screening tests

Table 2 reports sensitivity, specificity, PPV and NPV for the evaluated screening tests, individually (VIA, colposcopy, LBC⩾ASCUS, and HC2) and in set combinations (LBC using HC2 to triage ASCUS, and positivity of either LBC⩾ASCUS or HC2), estimated by both crude and corrected models. The corrected estimates included an additional 16.2 cases of CIN2+ imputed among women without biopsies (Appendix 1). Correction had minimal effect on estimates of sensitivity of HC2 alone, or of LBC⩾ASCUS or HC2, but sensitivity for VIA, colposcopy, LBC⩾ASCUS and HC2 triage of ASCUS decreased by 6–7%. Positivity for LBC⩾ASCUS or HC2 showed the highest sensitivity (95.5% corrected estimate), followed by HC2 alone (90.4%) and HC2 triage of ASCUS (84.0%). Corrected estimates of sensitivity for colposcopy and VIA were 54.1 and 37.1%, respectively. Corrected estimates of specificity ranged from 77.3% for LBC⩾ASCUS or HC2, up to 93.8% for HC2 triage of ASCUS. Visual inspection with acetic acid, colposcopy, LBC⩾ASCUS and HC2 all showed corrected specificity between 85.1 and 89.4%. Positive predictive value was highest for HC2 triage of ASCUS (33.1%), followed by HC2 (19.5%) and LBC⩾ASCUS (17.8%), all of which showed an NPV of at least 99.4%. Positive predictive value and NPV were lower for colposcopy (11.7 and 98.1%, respectively) and VIA (10.0 and 97.8%).

With respect to the 22 CIN3 cases, all were HC2-positive, 21 were LBC⩾ASCUS-positive and 10 were colposcopy-positive. Of the nine CIN3 cases arising in study sites where VIA was evaluated, four were VIA-positive. The only case of invasive cancer was an HC2-positive, but colposcopy-negative, high-grade squamous intraepithelial neoplasia.

**Figure 1:**
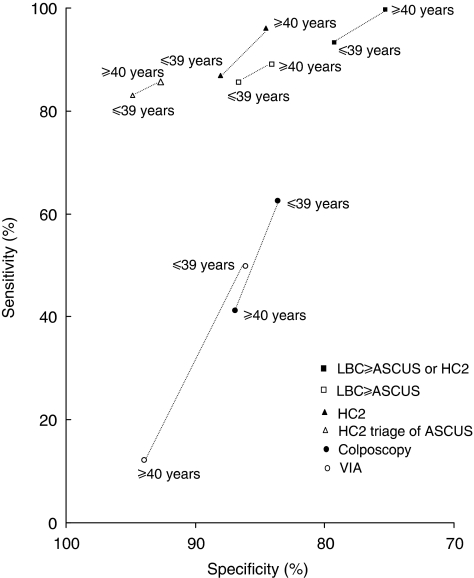
Sensitivity and specificity for different screening tests by age group. LBC⩾ASCUS=liquid-based cytology with a threshold of atypical squamous cells of undetermined significance; HC2=hybrid capture 2; VIA=visual inspection with acetic acid.

Figure 1 shows the comparison of screening test performance among women aged 39 years or younger and 40 years or older, based upon corrected estimates. Visual inspection with acetic acid was significantly more sensitive in women aged 39 years or younger (49.9%) than older women (12.2%; *χ*_1_^2^=7.51; *P*=0.006). Also colposcopy was slightly more sensitive, whereas LBC⩾ASCUS, HC2 and their combinations were slightly less sensitive in younger than older women, although none of these differences were statistically significant.

The sensitivity and specificity of different screening tests varied somewhat across study sites, but no significant heterogeneity emerged (data not shown). Furthermore, the performance of the screening tests relative to each other, most notably the relatively lower sensitivity of visual methods in comparison with LBC, HC2 and their combinations were consistent across the three study sites (data not shown).

## Discussion

In our present evaluation of different cervical cancer screening approaches in China, LBC, HC2 and their combinations performed well, whereas VIA missed a majority of CIN2+, particularly in older women.

Most cervical screening studies suffer, to a varying extent, from verification bias, especially due to the reliance on a visual method (colposcopy) as a gold standard, without the possibility for a positive result using morphological (cytology) and/or virological (HPV) methods to trigger disease evaluation in colposcopy-negative women (Mahé and Gaffikin, 2005). This is a particular problem for alternative visual methods, such as VIA, the results of which are closely correlated with those of colposcopy (Pretorius *et al*, 2007). In this study, verification bias was minimised through histological disease assessment being prompted by a positive result for either VIA, colposcopy or LBC (using HC2 to triage ASCUS). Furthermore, although no truly random biopsies were obtained, biopsies triggered by fluorescence spectroscopy, which proved to be a poor screening test, were used to estimate underlying CIN2+ in women negative for all the above tests.

Visual inspection with acetic acid is an inexpensive and simple screening approach that has been widely evaluated, although often with substantial verification bias (IARC, 2005). In this study, VIA was by far the least sensitive of the evaluated tests across the three study sites. Visual inspection with acetic acid sensitivity (37%) from our study fell into the lower range of earlier estimates (29–95%) (Belinson *et al*, 2001; Ferreccio *et al*, 2003; IARC, 2005; De Vuyst *et al*, 2005; Sarian *et al*, 2005; Sangwa-Lugoma *et al*, 2006; Almonte *et al*, 2007; Pretorius *et al*, 2007; Sankaranarayanan *et al*, 2007), even when compared only to those studies with minimal verification bias (50–71%, Belinson *et al*, 2001; Sarian *et al*, 2005; Sangwa-Lugoma *et al*, 2006; Almonte *et al*, 2007), and despite the fact that VIA was performed exclusively by gynaecologists rather than nurses (Sangwa-Lugoma *et al*, 2006). The specificity of VIA (90%) fared better in relation to the estimates from earlier studies (62–98%) (Belinson *et al*, 2001; Ferreccio *et al*, 2003; IARC, 2005; De Vuyst *et al*, 2005; Sarian *et al*, 2005; Sangwa-Lugoma *et al*, 2006; Almonte *et al*, 2007; Pretorius *et al*, 2007; Sankaranarayanan *et al*, 2007), suggesting that gynaecologists may have been more cautious to classify a women as VIA-positive in the present (11%) than in earlier studies (3–53%). Indeed, acetowhite lesions were classified as positive only when a distinct pattern was noted, the expected outcome being that the application of a less conservative criterion for VIA would increase sensitivity, with a corresponding drop in specificity (Denny *et al*, 2002; Sangwa-Lugoma *et al*, 2006).

An important factor in the relatively poor performance of VIA in our study was a strong decline in the sensitivity of the test in women aged 40 years or older. Some earlier studies have reported VIA sensitivity to be stable with age (Cronje *et al*, 2001; Sankaranarayanan *et al*, 2004a), but they suffered from verification bias. In a large study in Guanacaste, Costa Rica, the performance of cervicography, a variant of visual inspection with remote assessment of photographic images of the cervix, was noted to decrease substantially with age (Ferreccio *et al*, 2003). Additional evidence of the worse performance of VIA at older ages comes from a large randomised controlled trial in which VIA significantly prevented cervical cancer incidence and mortality in women aged 30–39 years, but not at older ages (Sankaranarayanan *et al*, 2007). These findings would suggest that although VIA may be the only screening approach practicable in many low-resource settings, it is most appropriate to invest in screening of women before they are of an age when the retreat of the transformation zone into the endocervix renders it invisible to visual methods (Sankaranarayanan and Wesley, 2003).

Colposcopy performed only moderately well as a primary screening test in our study, in agreement with suggestions from recent work (Jeronimo and Schiffman, 2006; Pretorius *et al*, 2007). Overall, sensitivity and specificity were only 54 and 85% respectively, which was similar to that in an earlier large study in China (Pretorius *et al*, 2007). Like VIA, colposcopy missed more than half of all CIN2+ among women aged 40 years or older. Colposcopy was substantially less sensitive than LBC or HC2, as in earlier studies with minimal verification bias (Belinson *et al*, 2001; Pretorius *et al*, 2007). The performance of visual methods appeared similar when analyses were restricted to CIN3.

Using ASCUS as a cutoff, LBC showed a sensitivity of 87% and a specificity of 85%, which is consistent with that seen in earlier evaluations (Belinson *et al*, 2001; Davey *et al*, 2006; Almonte *et al*, 2007). Liquid-based cytology was performed in two expert laboratories, and so its quality should not be considered as representative of some locally performed conventional cytology. The sensitivity of conventional cytology has been shown to be poorer than LBC in some low-resource settings (Ferreccio *et al*, 2003; Almonte *et al*, 2007), and often only equivalent to, or even worse than, that of VIA (Sankaranarayanan *et al*, 2004b; Sarian *et al*, 2005; Sangwa-Lugoma *et al*, 2006; Almonte *et al*, 2007).

Hybrid capture 2 sensitivity and specificity estimates were within the range of those from earlier studies (IARC, 2005; Sarian *et al*, 2005; Almonte *et al*, 2007), and similar to a pooled analysis including 25 studies (Koliopoulos *et al*, 2007). The use of HC2 to triage ASCUS resulted in a slight drop in sensitivity compared with LBC alone, but with a substantial gain in specificity over LBC or HC2 alone, so that the PPV (33.1%) was the best of all screening tests. Allowing either LBC⩾ASCUS or HC2 to determine test positivity increased the sensitivity, but resulted in a corresponding drop in specificity.

Strengths of our study included the large number and broad age range of participants who were drawn from three geographically and socioeconomically diverse areas in China. Screening tests were performed independently of each other and according to similar protocols in the study sites, and an invitation procedure based on population lists ensured the representativeness of the study populations. A limitation of this study was the number of women who were lost to follow-up before a histological confirmation of abnormal screening findings could be made. As a remedy, we envisaged a model that corrected for the lack of certain biopsies, but it is of note that these imputed biopsies were few and had relatively little impact on the conclusions of this study, which would not have differed substantially if crude estimates of sensitivity and specificity had been used.

Given their requirement for specialised infrastructure and relatively high cost, the potential application of LBC and/or HC2 to low-resource settings remains unclear. Given the robustness of LBC and HPV samples to both time and temperature, however, our study suggests the feasibility of sending specimens to centralised laboratories, which can bring down testing costs through economies of scale. Both tests can be automated and show good interlaboratory agreement. However, such an approach would still require additional visits for diagnostic work-up and treatment of women with positive tests, which is known to result in considerable losses to follow-up. The potential advent of a new rapid and inexpensive HPV DNA test requiring minimal laboratory infrastructure (Lorincz, 2006) would allow a see and treat approach similar to that currently possible for VIA, but with a potentially more accurate test.

## Figures and Tables

**Table 1:** Description of selected characteristics, screening test positivity and biopsies taken among study participants by study site

	**Shanxi**	**Liaoning**	**Guangdong**	**All**
	***N* (%)**	***N* (%)**	***N* (%)**	***N* (%)**
Total	743 (100)	699 (100)	1120 (100)	2562 (100)
				
*Selected characteristics*				
*Age group (years)*				
15–29	147 (19.8)	121 (17.3)	348 (31.1)	616 (24.0)
30–39	198 (26.7)	193 (27.6)	384 (34.3)	775 (30.3)
40–49	197 (26.5)	191 (27.3)	295 (26.3)	683 (26.7)
50–59	201 (27.1)	194 (27.8)	93 (8.3)	488 (19.1)
				
Secondary education	86 (11.6)	317 (45.4)	895 (79.9)	1298 (50.7)
Unmarried	26 (3.5)	76 (10.9)	151 (13.5)	253 (9.9)
>1 lifetime sexual partner	129 (17.4)	117 (16.7)	167 (14.9)	413 (16.1)
Pap smear history	68 (9.2)	45 (6.4)	232 (20.7)	345 (13.5)
				
*Screening test outcomes*				
VIA positive	Not assessable	65 (9.3)	142 (12.7)	207 (11.4)
Colposcopy positive	165 (22.2)	105 (15.0)	146 (13.0)	416 (16.2)
LBC⩾ASCUS positive	138 (18.6)	76 (10.9)	225 (20.1)	439 (17.1)
HC2 positive	119 (16.0)	144 (20.6)	155 (13.8)	418 (16.3)
Biopsies taken	301 (40.5)	232 (33.2)	120 (10.7)	653 (25.5)

VIA=visual inspection with acetic acid; LBC⩾ASCUS=liquid-based cytology with a threshold of atypical squamous cells of undetermined significance; HC2=hybrid capture 2.

**Table 2:** Crude and corrected sensitivity, specificity, PPV and NPV for CIN2+, by test

		**Sensitivity**		**Specificity**		**PPV**		**NPV**	
**Tests**	**Status**	**%**	**95% CI**	**%**	**95% CI**	**%**	**95% CI**	**%**	**95% CI**
VIA	Crude	42.9	(27.7–59.0)	89.4	(87.8–90.8)	8.7	(5.2–13.4)	98.5	(97.8–99.0)
	Corrected	37.1	(26.4–49.3)	89.4	(87.9–90.8)	10.0	(6.7–14.6)	97.8	(97.1–98.4)
									
Digital colposcopy	Crude	60.8	(48.8–72.0)	85.1	(83.6–86.5)	10.8	(8.0–14.2)	98.6	(98.1–99.1)
	Corrected	54.1	(44.6–63.3)	85.1	(83.7–86.5)	11.7	(9.0–15.1)	98.1	(97.5–98.5)
									
LBC⩾ASCUS	Crude	93.2	(84.9–97.8)	85.1	(83.7–86.5)	15.7	(12.4–19.5)	99.8	(99.5–99.9)
	Corrected	86.9	(81.1–91.1)	85.4	(84.0–86.7)	17.8	(14.7–21.5)	99.4	(99.2–99.6)
									
HC2	Crude	90.5	(81.5–96.1)	85.9	(84.5–87.2)	16.0	(12.6–19.9)	99.7	(99.3–99.9)
	Corrected	90.4	(83.3–94.7)	86.4	(85.0–87.7)	19.5	(16.3–23.2)	99.6	(99.3–99.8)
									
HC2 triage of ASCUS	Crude	90.5	(81.5–96.1)	93.5	(92.4–94.4)	29.3	(23.5–35.6)	99.7	(99.4–99.9)
	Corrected	84.0	(77.4–88.9)	93.8	(92.8–94.7)	33.1	(27.6–39.0)	99.4	(99.1–99.6)
									
LBC⩾ASCUS or HC2	Crude	95.9	(88.6–99.2)	76.9	(75.2–78.5)	11.0	(8.7–13.7)	99.8	(99.5–100.0)
	Corrected	95.5	(90.0–98.1)	77.3	(75.7–78.9)	13.3	(11.1–15.9)	99.8	(99.5–99.9)

PPV=positive predictive value; NPV=negative predictive value; CI=confidence interval; CIN2+=cervical intraepithelial neoplasia grade 2 or worse; VIA=visual inspection with acetic acid; LBC⩾ASCUS=liquid-based cytology with a threshold of atypical squamous cells of undetermined significance; HC2=hybrid capture 2.

**Table A1:** Number of CIN2+ confirmed and estimated among women with and without biopsies, respectively, by combination of LBC, HC2 and digital colposcopy results

**LBC**	**HC2**	**Digital colposcopy**	**Women with biopsies**		**Women without biopsies**		**Total CIN2+**	
			***N***	**Confirmed CIN2+**	***N***	**Estimated CIN2+**	***N***	**%**
Normal	−	−	172	0	1482	0.0	0.0	0.0
ASCUS	−	−	22	0	158	0.0	0.0	0.0
LSIL	−	−	9	0	2	0.0	0.0	0.0
HSIL	−	−	1	0	0	0.0	0.0	0.0
Normal	−	+	194	3	68	1.1	4.1	1.5
Normal	+	−	28	1	153	5.5	6.5	3.6
Normal	+	+	20	1	6	0.3	1.3	5.0
ASCUS	−	+	23	2	7	0.6	2.6	8.7
ASCUS	+	−	50	10	18	3.6	13.6	20.0
LSIL	−	+	5	1	0	0.0	1.0	20.0
ASCUS	+	+	37	8	1	0.2	8.2	21.6
LSIL	+	−	23	6	10	2.6	8.6	26.1
LSIL	+	+	34	18	3	1.6	19.6	52.9
HSIL	+	+	17	11	0	0.0	11.0	64.7
HSIL	+	−	17	12	1	0.7	12.7	70.6
HSIL	−	+	1	1	0	0.0	1.0	100.0
								
Total			653	74	1909	16.2	90.2	3.5

CIN2+=cervical intraepithelial neoplasia grade 2 or worse; LBC=liquid-based cytology; HC2=hybrid capture 2; ASCUS=atypical squamous cells of undetermined significance; LSIL=low-grade squamous intraepithelial lesion; HSIL=high-grade squamous intraepithelial lesion.

## Appendix 1

Table A1
